# Inflammation and Obesity: The Pharmacological Role of Flavonoids in the Zebrafish Model

**DOI:** 10.3390/ijms24032899

**Published:** 2023-02-02

**Authors:** Caterina Russo, Alessandro Maugeri, Laura Musumeci, Giovambattista De Sarro, Santa Cirmi, Michele Navarra

**Affiliations:** 1 Department of Chemical, Biological, Pharmaceutical and Environmental Sciences, University of Messina, 98166 Messina, Italy; 2 Department of Health Sciences, University “Magna Græcia” of Catanzaro, 88100 Catanzaro, Italy

**Keywords:** flavonoids, zebrafish, *Danio rerio*, obesity, inflammation, metabolic disorders, overweight, natural products, diet, polyphenols

## Abstract

A Mediterranean-style diet is highly encouraged thanks to its healthy food pattern, which includes valuable nutraceuticals such as polyphenols. Among these, flavonoids are associated with relevant biological properties through which they prevent or fight the onset of several human pathologies. Globally, the enhanced incidence of overweight and obese people has caused a dramatic increase in comorbidities, raising the need to provide better therapies. Therefore, the development of sophisticated animal models of metabolic dysregulation has allowed for a deepening of knowledge on this subject. Recent advances in using zebrafish (*Danio rerio*) as model for metabolic disease have yielded fundamental insights into the potential anti-obesity effects of flavonoids. Chronic low-grade inflammation and immune system activation seem to characterize the pathogenesis of obesity; thus, their reduction might improve the lipid profile of obese patients or prevent the development of associated metabolic illnesses. In this review, we highlight the beneficial role of flavonoids on obesity and related diseases linked to their anti-inflammatory properties. In light of the summarized studies, we suggest that anti-inflammatory therapies could have a relevant place in the prevention and treatment of obesity and metabolic disorders.

## 1. Introduction

The Mediterranean diet is considered a reliable tool to counteract inflammation and prevent related pathologies, with fruit and vegetables as its main weapons [1]. They represent a valuable food source of bioactive molecules, mostly flavonoids, endowed with a broad spectrum of well-recognized pharmacological properties including antioxidant, [2] anti-inflammatory [3], immunomodulatory [4], neuroprotective [5,6], and antitumoral [7,8,9].

Zebrafish (*Danio rerio*) is a well-consolidated model, useful in the research of potentially active molecules in the prevention and in the treatment of several diseases. In particular, the advances in the establishment and new applications of the zebrafish model encouraged its use in assessing both the bioactivity and safety of substances from the plant kingdom, including flavonoids [10,11]. Interestingly, zebrafish straddles the line between in vitro and murine models, rapidly and economically providing an insight into the whole organism. They present the key organs and a control system of metabolic processes comparable to those in humans, thus proving to be a suitable model for the study of metabolic disorders [12].

It is known that dietary imbalances can alter the equilibrium between the intake, utilization, and storage of energy, leading to an increase in fat reserves, as well as triglyceride and cholesterol levels in the blood. These events, along with a sedentary lifestyle, smoking habits, and the presence of genetic factors can contribute to the development and progression of metabolic disorders including obesity [13]. In recent years, the prevalence of overweight people or those affected by obesity recorded pandemic levels (more than 1.9 billion people worldwide [14]), causing a dramatic increase in comorbidities, such as diabetes, cardiovascular complications, inflammatory bowel disease, and cancer [15,16,17,18]. The prevention and treatment of this pathology remain complex. To date, therapy for obesity consists of drugs acting as appetite suppressants, gastric emptying, satiety promoters, or energy expenditure, as well as modulators of insulin and glucagon secretion or inhibitors of pancreatic and gastric lipases. Unfortunately, all of these agents have been associated with serious side effects, such as anxiety, hypertension, and severe gastrointestinal disorders, which limit their efficacy and the compliance of patients [19,20]. On the contrary, the use of bariatric devices and surgical procedures is usually the least effective strategy to fight obesity [21].

Dietary modifications consisting of a high intake of fruit and vegetables have been suggested to prevent or counteract the rise of metabolic disorders [22]. In this regard, flavonoids have been shown to regulate several molecular pathways and exert potentially beneficial effects in the prevention and treatment of obesity and related diseases by acting via multiple mechanisms [23].

In this review, we have collected the most relevant studies on the anti-obesity effects induced by flavonoids in zebrafish models, shedding light on the link existing between inflammation and obesity.

## 2. Obesity and Related Diseases

The World Health Organization (WHO) defined obesity as one of the main health concerns of the 21st century. This is because 39% of adults worldwide are overweight (body mass index -BMI- ≥ 25 kg/m^2^) and 13% are obese (BMI > 30 kg/m^2^), thus representing a serious issue for global public health [14]. It develops as a result of the imbalance between energy intake and expenditure, or between the lipogenesis and lipolysis processes, which causes fat accumulation and the dysfunction of adipose tissue characterized by an increase in the number and size of adipocytes, known as “hyperplasia” and “hypertrophy”, respectively.

In physiological conditions, energy metabolism is regulated by adipocytokines secreted from adipocytes, such as hormones (i.e., leptin, adiponectin, resistin, etc.) and cytokines (i.e., interleukin -IL-6, IL-10, and tumor necrosis factor -TNF-α) [24]. On the contrary, excess nutrients and the hypertrophy of adipocytes trigger an inflammatory process in adipose tissue via the activation of proinflammatory mediators (i.e., nuclear factor kappa B -NF-κB-, IL-1β, IL-6, TNF-α, C-reactive protein -PCR-, monocyte chemoattractant protein -MCP-1, mitogen-activated protein kinases -MAPKs-, etc.) [25,26,27], thus determining a consequent deceleration of the metabolic rate. Interestingly, high levels of fatty acids also activate the NLRP3 inflammasome in macrophages infiltrating the adipose tissue of obese people, causing a local inflammation that further impairs the adipogenesis process [28]. Moreover, the saturation of fat reserves occurring in adipose tissue determines an increase in lipid flux toward other tissues or organs, such as the liver and muscle. Here, fat accumulation causes a phenomenon of lipotoxicity, responsible for increased activation of autophagy and apoptotic processes, a high number of infiltrating immune cells, and an altered expression of tissue-specific proteins (i.e., perilipins -PLINs-, peroxisome proliferator-activated receptors -PPARs-, etc.) [29,30,31]. This fatty acid excess in body districts other than adipose tissue can also impair insulin action or cause non-alcoholic fatty liver disease (NAFLD), meaning a spectrum of liver tissue changes ranging from steatosis to non-alcoholic steatohepatitis (NASH), cirrhosis, and eventually, possibly leading to hepatocellular carcinoma [32].

The gut microbiota alterations, known as dysbiosis, can also contribute to the onset and development of obesity. A high presence of bacteria strains Firmicutes (Gram-positive) and fewer Bacteroidetes (Gram-negative) was observed in animal models and humans [33]. Alternatively, inflammation-related obesity may result from an increase in lipopolysaccharide (LPS)-producing bacteria [34] and intestinal permeability.

Obesity represents the main risk factor for the development of metabolic diseases underlying an inflammatory mechanism, such as fatty liver disease, commonly known as hepatic steatosis, hyperlipidemia, and insulin resistance [32]. Again, obesity may underlie a more complex cluster of diseases, namely metabolic syndrome, which also includes hyperglycemia, hypertriglyceridemia, and hypertension [35].

In this light, multiple approaches must be considered in treating obesity and preventing associated pathologies, such as exploiting the anti-inflammatory potential of some compounds. Therefore, the role of inflammation in obesity is worthy of clarification.

As already known, inflammation arises in a protective response to tissue or organ damage, triggering a sequence of events aimed at restoring the original functionality of the affected part via the destruction of the injurious agents. The five hallmarks of the inflammatory process are calor, rubor, tumor, dolor, and functio laesa, and, depending on its duration, inflammation can be distinguished as acute or chronic.

In obese subjects, the excess of macronutrients predisposes the organism to a proinflammatory state and oxidative stress. This explains the increase in levels of inflammatory mediators IL-6 and TNF-α and the reduction in anti-inflammatory markers such as adiponectin observed in adipose tissues as well as the secretion of PCR by the liver [36]. Moreover, when this inflammatory state is followed by endothelial dysfunction, a lowering of nitric oxide levels and an increase in reactive oxygen species are observed, thus generating an oxidative stress condition. Therefore, the documented presence of inflammatory indicators demonstrated that obesity implies a chronic low-grade inflammation and activation of the immune system [37,38].

However, inflammation is an initiation mechanism for developing cardiovascular (atherosclerosis and coagulation) and metabolic (obesity, metabolic syndrome, and diabetes) diseases as well as neurodegenerative processes, osteoporosis, and cancer [36]. Notably, the inflammatory status which appears during the aging process accelerates the onset of the abovementioned inflammatory- and age-based diseases [39]. Therefore, systemic inflammatory markers can be predictors of the development of pathologies, such as obesity.

## 3. Zebrafish as a Model of Obesity and Metabolic Disorders: Advantages and Disadvantages

The basal pathogenesis of obesity is not yet fully understood, hence the use of animal models appear functional in improving knowledge on this illness and in identifying new effective treatments.

Zebrafish have been revealed as an appealing model for studying some diseases, especially obesity and metabolic disorders [40]. They are vertebrates possessing high genetic, anatomical, and physiological similarities to humans. Moreover, they have the key organs implied in metabolic processes, including digestive organs, adipose tissue, liver, and skeletal muscle. Very similar to the human counterpart, they also conserve some key functions such as appetite regulation in the brain, the control of insulin release, and lipid storage as well as metabolic pathways (i.e., PPARs, sterol regulatory element-binding proteins -SREBPs-, liver X receptors -LXRs-) involved in adipocyte differentiation, energy homeostasis, and cholesterol metabolism [41,42]. Furthermore, the involvement of the brain-derived neurotrophic factor (BDNF)/tropomyosin receptor kinase B (TrkB) system in the regulation of food intake and energy balance has also been observed in zebrafish as in mammals [43].

Notably, zebrafish respond well to diet modifications, modulating the leptin and adiponectin levels and the expression of their receptors [44]. Similar to humans, an excessive intake of nutrients causes high triglyceride levels and hepatic steatosis in zebrafish. Again, in this model, lipids are accumulated both in visceral, intramuscular, and subcutaneous adipose tissue, thus allowing us to examine the body fat distribution in its entirety. Both the conservation, distribution, and formation of fat in the adipose tissue of zebrafish are comparable to those of mammals, and the optical transparency of zebrafish enables monitoring, in vivo, adipocyte formation, and fatty acid uptake in a temporal manner [45]. Remarkably, the characteristic external development of zebrafish makes it easy to perform embryonic and genetic manipulations and rapidly obtain genetic information. They share 67–74% identity in the genome for PPARs with mice and humans, resulting in a promising model to investigate the role of these receptors in adipogenesis and obesity [42]. In addition, it was seen that microbiota can be easily modulated in zebrafish [46].

However, zebrafish are ectotherm species whose metabolism is not regulated by temperature, and, therefore, they lack brown adipose tissue (BAT). Some genes of lipid metabolism are not fully conserved according to human sequence and function. For example, zebrafish leptin is only 19% similar to human leptin and it is not expressed in the adipose tissue of zebrafish, which also lack leptin receptors [47]. Similarly, uncoupling proteins (UCPs) are localized in districts such as the brain, liver, and muscle tissue, but unlike humans, they are not present in the adipose tissue of zebrafish [48]. Moreover, the lack of protocols standardizing feeding and housing conditions still represents a great limitation in the use of these models. Overall, the low maintenance cost, a short life cycle, and the availability of various gene-editing tools, make zebrafish a model more suitable for large-scale experiments than bigger animal models (i.e., dog, pig, and nonhuman primates). However, zebrafish are a nonmammalian model, hence their translational value is limited compared to that of murine models (i.e., rodents and laboratory mice), which are closer to humans in terms of anatomy and, especially, physiology [49]. On the contrary, zebrafish share, with vertebrates, organ composition, cellular types, metabolic processes, and 70% of human orthologous genes. In this view, zebrafish are considered a good intermediate tool between in vitro and murine models, by projecting a clear image from a whole organism in a fast and economic manner [11].

To date, the current models of obesity employing zebrafish are obtained through the administration of a high-fat diet, or over-nutrition, by gene manipulation and the modification of gut microbiota composition [46]. In addition, adipocytes and fat droplets can be visualized in zebrafish larvae by staining with Oil Red O and Sudan dyes, as well as fluorescent colorants such as Nile Red and Lipid Green. This model lends itself to the use of fluorescent probes to monitor lipid metabolism, as well as to the application of subcellular resolution methods to observe the whole organism [50].

Finally, the cost-effectiveness of zebrafish breeding, the optical transparency, and the rapid reproduction rate make this animal model worthy of consideration for also studying other diseases, especially neurodegenerative and cardiovascular disorders, infections, and cancer [51,52,53,54]. In this setting, advanced approaches in the field of microscopy, transcriptomics, and metabolomics have succeeded in providing fundamental insights into the mechanisms of diseases using a zebrafish experimental model [55].

All of the advantages and limitations of the zebrafish model are summarized in Table 1.

## 4. Flavonoids and Inflammation in Obesity

**Figure 1:**
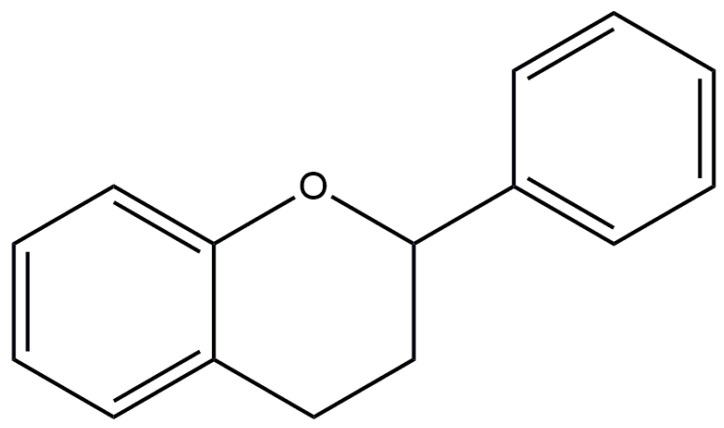
The basic skeleton of flavonoids.

Numerous polyphenolic compounds have been identified in plants, among which flavonoids represent the most important class. They are secondary plant metabolites, widely distributed in fruits and vegetables and their derivates such as wine, juices, and beer. Structurally, flavonoids consist of an aromatic ring joined to an oxygenated heterocyclic ring linked to a phenyl group. Depending on the degree of saturation of the heterocyclic ring, the number of oxygenated substituents, and the position of the phenyl group, flavonoids are classified into flavones, flavonols, flavanones, isoflavones, and anthocyanins (Figure 1). Of note, the flavonoid structure provides relevant metal chelation properties, conferring to these compounds a great antioxidant power [56]. Indeed, they have proven to exert protective effects against oxidative damage and related diseases [57]. More recent studies have identified new molecular pathways that deepen the knowledge of the anti-inflammatory activity of flavonoids [58,59] and anti-obesity pathways [23]. Interestingly, an inverse relationship between flavonoid intake and the risk of obesity has been recently proposed, which may be ascribed to the flavonoid effect on obesity-related inflammation. In this regard, an increased flavonoid assumption has been associated with a lowering effect on BMI and PCR levels, commonly produced in response to an inflammatory state [60]. On this line, adherence to a healthy diet rich in plant foods endowed with anti-inflammatory properties has been recently associated with a lower risk of obesity [61].

Notably, quercetin showed to inhibit the obesity-associated inflammatory response via the adenosine monophosphate-activated protein kinase (AMPK) phosphorylation and the activation of sirtuin 1 (SIRT1) in macrophages [62], sharing the same mechanism of action as other flavonoids [63]. Besides SIRT1, other human sirtuins showed to be a potential target of flavonoids [64,65]. Moreover, quercetin also reduced obesity-induced hepatic inflammation by promoting a switch in the macrophage phenotype [66], whereas naringenin was able to reduce the number of neutrophils infiltrating the adipose tissue of obese mice [67]. Other studies demonstrated a reduction in adipocyte inflammation via decreasing the toll-like receptor (TLR)-4 expression by epigallocatechin-3-gallate (ECGC) [68] and the modulation of PPARα/γ and c-Jun N-terminal kinase (JNK) pathways by daidzein [69].

Therefore, considering their anti-inflammatory effects, it appears evident that flavonoids can reduce obesity-related inflammation. Moreover, the anti-obesity potentiality of flavonoids is also reflected in the control of appetite, the reduction in food intake and intestinal fat absorption, on the regulation of metabolic processes (i.e., adipocyte differentiation, adipogenesis, lipolysis, and β-oxidation), on the induction of no-shivering thermogenesis, on the stimulation of energy expenditure, and the modulation of gut microbiota [23]. In recent years, zebrafish were found to be a model suitable for defining the mechanism of action of flavonoids in obesity and related diseases, even shedding light on inflammation as a possible central driver in the development and treatment of this metabolic disorder.

### 4.1. Anti-Obesity Effects of Flavonoids in Zebrafish Model

Lipid metabolism plays a key role in regulating inflammation within the context of acute and chronic diseases, such as cardiovascular, obesity, and metabolic disorders. In this regard, nutritional and therapeutic approaches targeting lipid metabolism have been suggested as promising strategies to mitigate inflammation related to these pathologies [70]. A polyphenolic extract obtained from the by-product of wine lees of the Tempranillo grape variety, in which rutin and quercetin are the major components, was revealed to be a potential food ingredient for weight management, modulating lipid metabolism in zebrafish. In particular, a 40% reduction in the fat reserves of fish embryos was facilitated, altering the expression of genes involved in lipid transport (microsomal triglyceride transfer protein -MTP, *mtp*), lipogenesis (fatty acid synthase -FASN, *fasn*), and β-oxidation (carnitine palmitoyl transferase 1B -CTP1B, *ctp1b*). It also induced the remodeling of the fatty acid levels in the phospholipid and triglyceride fractions and a reduction in the content of trans-oleic and stearic fatty acids [71]. Another polyphenolic extract obtained from the juice of white grapes (*Vitis vinifera),* namely WGJe, was displayed to counteract the weight gain in the zebrafish model. Pretreatment with WGJe lowered the BMI values, reduced the area of adipose tissue (both at subcutaneous and visceral levels), and decreased the number and the size of adipocytes in overfed zebrafish. This effect, at least in part, can be traced back to the well-known anti-inflammatory property of bioactive compounds present in WGJe at the adipose tissue level [72,73]. Moreover, WGJe exhibited anti-obesity effects also at the gene level, by restoring the altered expression of ghrelin (*ghrl*) and leptin (*lep*) in obese fish [74]. Noteworthily, the beneficial effects of grape extracts against obesity pathogenesis can be associated with their content in flavonoids and phenolic acids. This is because these components possess significant pharmacological properties [75,76,77] that could jointly contribute to improve the management of obesity and its associated comorbidities.

A blockage of lipid accumulation was even induced by flavonoid silibinin, mainly found in milk thistle (*Silybum marianum*). Interestingly, silibinin suppressed adipogenesis in its early stage and also triglyceride accumulation by downregulating the expression of adipogenic factors (PPARγ -*pparg*-, CCAAT-enhancer-binding protein alpha -C/EBPα, *cebpa*-, and fatty-acid-binding protein 4 -FABP4, *fabp11a*) in 3T3-L1 cells and the zebrafish homologs [78]. Similarly, baicalein, a flavonoid from *Scutellaria baicalensis*, exhibited an anti-adipogenic effect in zebrafish fed with a high-fat diet by significantly decreasing their lipid accumulation by over 30%. This effect appears ascribable to a diminution of the expression of adipogenic genes (*pparg*, *cebpa*, adipocyte protein 2 a/b -aP2-a/b, *fabp2a/b*-, and SREBP-1/2 -*srebf1/2*) induced by baicalein in a dose-dependent manner [79]. However, among flavones, baicalein has gained major attention for its potent inhibitory effect on the inflammatory response by modulating the expression of cytokines and related genes in numerous pathological conditions such as metabolic disorders [80].

Again, kaempferol, a flavonoid contained in *Kaempferia galanga* and *Opuntia ficus indica* var. *saboten* inhibited fat accumulation in diet-induced obese zebrafish. Nile Red staining allowed the observation of a reduction in the number of intracellular lipid droplets in obese zebrafish treated with kaempferol which, like baicalein, is due to a downregulation of the abovementioned adipogenic genes [81]. However, a relevant anti-obesity potentiality of kaempferol results from its anti-inflammatory and modulatory properties of gut microbiota already documented in vivo [82], but not yet in the zebrafish model. In this regard, an inhibitory effect of quercetin, a flavonoid mainly isolated from *Houttuynia cordata*, on obesity-induced inflammation was observed in 3T3-L1 and RAW264.7 cells, zebrafish, and mouse models. In vitro, quercetin inhibited MAPK signaling factors, extracellular signal-regulated kinase 1/2 (ERK_1/2_), JNK, and p38, involved in both inflammation and adipogenesis, as well as the adipokines MCP-1 and TNF-α, attracting macrophages into adipose tissue. It also counteracted the secretion of inflammatory cytokines IL-1β and IL-6 and stimulated the release of IL-10 anti-inflammatory cytokine. This anti-inflammatory mechanism of quercetin also likely occurred in zebrafish, where a decreased lipid accumulation and nitric oxide (NO) generation was observed in the quercetin-treated group compared to the untreated group. These data have been confirmed in mice, where quercetin lowered the body weight by almost 40% and suppressed the expression of adipogenic (C/EBPα, PPARγ, and FABP4), lipogenic (mammalian target of rapamycin -mTOR- and p70 ribosomal protein S6 kinase -p70S6K-), and inflammatory cytokines (TNF-α, IL-1β, and IL-6) [83].

Among the richest food sources of flavonoids, citrus juices, and their derivatives are in the lead, with their acknowledged antioxidant and anti-inflammatory effects [84,85] potentially exploited to counteract inflammation-based diseases such as obesity [86]. In this regard, a flavonoid-rich extract of orange juice (OJe) has been shown to significantly reduce body weight and BMI values in overfed zebrafish. In addition, OJe decreased the number and size of adipocytes, in the visceral and subcutaneous adipose tissues of obese zebrafish. In the latter experimental model, it also stimulated the gene expression of anorexigenic signals (*lep* and pro-opiomelanocortin -POMC, *pomc*) and inhibited the orexigenic signals (*ghrl*, orexin -*hcrt*-, and neuropeptide Y -NPY, *npy*), both at intestinal and cerebral levels, similarly to another natural compound studied, namely melatonin [87]. On one hand, nobiletin and hesperidin flavonoids present in OJe appear, at least partly, responsible for the anti-obesity and lipolytic observed effects. On the other hand, the well-known link between inflammation and obesity [38], and even more well known, the in vivo evidence of anti-phlogistic properties of the whole extract [88], support an anti-inflammatory mechanism underlying the OJe effect against weight gain [89]. The neuromodulatory activity of OJe [90] and its influence on orexigenic and anorexigenic signals also accounted for the anti-obesity effect observed in zebrafish. Interestingly, some flavonoids have been shown to act on the brain–gut axis [91] and, like OJe, exert a central regulation of appetite and a consequent reduction in food intake in the gut [23]. Again, isoflavones, such as formononetin and ononin may turn out to be good candidates in obesity management, considering their potent anti-inflammatory effect on the LPS-stressed zebrafish models, via reducing the expression of proinflammatory cytokines and targeting myeloid differentiation primary response 88 (MyD88) or TIR-domain-containing adapter-inducing interferon-β (TRIF) ERK/JNK pathways [92]. This could also be the case with fisetin since it counteracted the LPS-induced inflammatory response and endotoxic shock in zebrafish larvae by suppressing the crosstalk between glycogen synthase kinase 3β (GSK-3β)/β-catenin and the NF-κB signaling pathways [93]. Instead, the flavone wogonin drove inflammatory neutrophils toward apoptosis in the zebrafish model of sterile tissue injury. This mechanism of action, also shared in vitro by luteolin and apigenin, might be the prelude to the discovery of a novel class of anti-inflammatory agents, useful in treatment of human inflammatory diseases [94].

Table 2 summarizes the anti-obesity properties of the flavonoids discussed in this section.

### 4.2. Role of Flavonoids on Obesity-Related Inflammatory Diseases in Zebrafish

There is a strong correlation between obesity and the development of NAFLD, commonly known as hepatic steatosis [95]. In this line, the strongest antioxidant flavonoid of *Citrus limon*, eriocitrin (eriodictyol 7-rutinoside), showed to exert protective effects against diet-induced hepatic steatosis in the zebrafish model. Indeed, the oral administration of eriocitrin ameliorated dyslipidemia and reduced lipid droplets in the liver via the activation of mitochondrial biogenesis. In particular, eriocitrin increased mitochondrial size and mtDNA content, thus promoting ATP production in induced diet-obese zebrafish [96]. Similarly, isobavachalcone (IBC), isolated from *Angelica keiskei*, reduced intrahepatic fat deposits and improved liver steatosis in zebrafish fed with a high-fat cholesterol diet [97], downregulating the expression of the genes *cebpa* and *pparg*. Naringenin (NAR), a predominant flavanone in *Citrus paradisi*, as well as its glycosylated derivative naringin (NRG), protected against inflammatory injuries such as alcoholic liver disease (ALD) by reducing lipid accumulation, apoptosis, and DNA damage in zebrafish larvae [98,99]. Similarly, the main flavonoids of *Puerariae Lobatae* radix (PLR), daidzein, and its glycosidic counterpart reduced fat accumulation, levels of cholesterol, and triglycerides in a zebrafish model of ALD. This effect was due to a downregulation of genes involved in lipid metabolism (cytochrome P450 CYP2y3 and CYP3a65, alcohol dehydrogenase ADH8a and ADH8b, 3-hydroxy-3-methyl-glutaryl-coenzyme A reductase -HMGCRB-, and FASN), in endoplasmic reticulum stress and DNA damage (C/EBP homologous protein -CHOP-, ER degradation-enhancing α-mannosidase-like protein -EDEM1-, growth arrest and DNA damage-inducible alpha -GADD45αa-, and activating transcription factor 6 -ATF6-) and a reduction in proinflammatory cytokines (IL-1β and TNF-α), by targeting the AMPKα/acetyl-CoA carboxylase (ACC) signaling pathway [100]. Notably, when liver injury occurs, different inflammatory cytokines are released by the P2X7 receptor in immune cells, causing inflammation. In this setting, quercetin significantly downregulated the expression of P2X7R, activating the phosphatidylinositol-3-kinase (PI3K)/Kelch-like ECH-associated protein 1 (Keap1)/nuclear factor erythroid-2–related factors 2 (Nrf2) axis in zebrafish transgenic larvae [101]. Another citrus flavonoid, nobiletin, reduced the expression of hepatic angiopoietin-like protein 3 (ANGPTL3), which regulates the lipid metabolism in high-fat diet-fed zebrafish. In the same model, it also lowered the plasma levels of triglycerides and cholesterol, revealing it as a potential protective agent for dyslipidemia and atherosclerotic cardiovascular diseases [102].

It is well known that intestinal health status is often impaired in cases of obesity, therefore the anti-inflammatory properties of citrus fruits and their flavonoids can be a help in the management of bowel diseases. In this regard, the pretreatment with a flavonoid-rich extract of orange juice (OJe) was able to counteract the inflammation of intestinal mucosa induced by *Vibrio anguillarum* in adult zebrafish, thus preventing enteritis [103]. Similarly, a polyphenol extract (CSVP) obtained from the mixture of *Castanea sativa* shell, an agri-food waste, and *Verbascum macrurum*, rich in tannins and flavonoids, prevented intestinal inflammation induced by k-carrageenan in the zebrafish model, downregulating proinflammatory markers (TNF-α -*tnfa*-*,* prostaglandin-endoperoxide synthase 2a -*ptgs2a*- and IL-1β -*il1b*) and upregulating the anti-inflammatory IL-10 (*il10*). Moreover, this extract also promoted the activation of MAPK signaling factors, leading to the suppression of the NF-κB pathway [104]. Like other agri-food waste, such as those from citrus processing [105], CSVP was revealed to be an interesting renewable source of bioactive molecules. The flavonoids (linarin, diosmetin-7-glucoside, and tilianin), phenolic acids, and polysaccharides of the chrysanthemum stem and leaf extract ameliorated the inflammatory bowel disease in zebrafish larvae exposed to sodium dextran sulfate (DSS), by regulating the expression of *il1b*, IL-8 (*il8*) and matrix metallopeptidase 9 (MMP-9, *mmp9*), and stimulating the superoxide dismutase activity [106]. Finally, *Coreopsis lanceolata* flowers and their isolated flavonoids (leptosidin, leptosin, isoquercetin, and astragalin) protected the pancreatic islets damaged by alloxan in zebrafish, thanks to their antioxidant and anti-inflammatory effects, thus also supporting the role of the inflammatory response in the pathogenesis of insulin resistance and type 2 diabetes [28,107].

**Figure 2:**
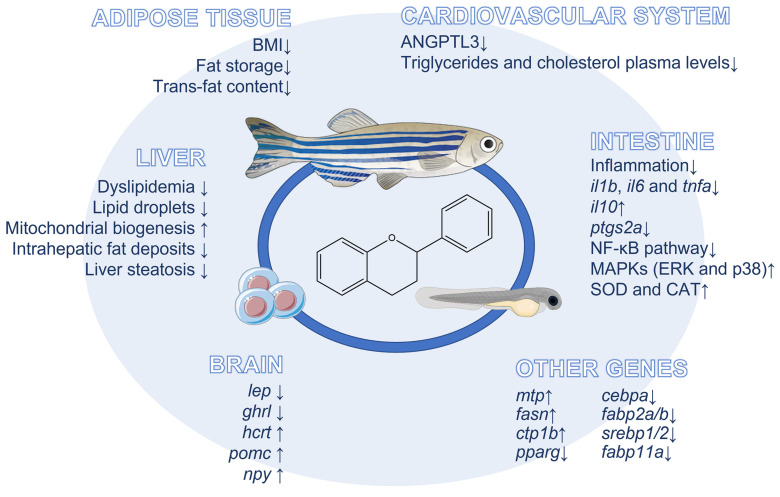
Mechanisms underlying the anti-obesity effects of flavonoids in the zebrafish model.

The abovementioned beneficial effects of flavonoids against obesity-related diseases observed in zebrafish models are reported in Table 3. Mechanisms underlying the beneficial effects of flavonoids both against obesity and related diseases observed in the zebrafish model are depicted in Figure 2.

## 5. Conclusions

The zebrafish model is an emerging model in the study of deregulated metabolism-driven diseases, such as obesity and related pathologies. Indeed, the versatility of using zebrafish, coupled with recent techniques in advanced microscopy, and transcriptomic and metabolomic analysis, allows for acceleration in the understanding of metabolic processes and their linked disorders. Considering the widespread presence and the well-known efficacy of natural products such as flavonoids, they are worthy of being evaluated in the management of obesity and associated comorbidities. Flavonoids affect various stages of the inflammatory process; therefore, their use might be a good strategy to counteract chronic low-grade inflammation, characterizing obesity and metabolic diseases. For the first time, we focused on the link between inflammation and obesity, highlighting the role of this class of plant secondary metabolites in the most suitable experimental model for metabolic disorders, such as zebrafish. Indeed, experimental data collected in this review support the beneficial activity of flavonoids on obesity, reflected in several mechanisms including an important anti-inflammatory effect. This is because the emerging strategy for the treatment of obesity increasingly aims to consider the disease as a whole and not as the sum of its parts, suggesting a multi-targeted approach. However, the pharmacokinetics of certain flavonoids limits their efficacy, so novel methods to improve the bioavailability of these molecules should be investigated. Deeper knowledge of the safety profile and drug interactions associated with flavonoids could help to better define the role of these compounds in obesity. Therefore, further investigations are fundamental to the continuation of the path toward the effective management of overweight and obesity.

## Figures and Tables

**Table 1:** Advantages and disadvantages of using zebrafish as a model of obesity and metabolic disorders.

**ADVANTAGES**
– Genetic, anatomical, and physiological similarities with humans
– Possess key organs of metabolic processes (digestive organs, adipose tissue, liver, and skeletal muscle)
– Conservation of key metabolic functions (appetite regulation in the brain, control of insulin release, and lipid storage)
– Conservation of metabolic pathways (i.e., PPARs, SREBPs, LXRs, and BDNF/TrkB system) involved in adipocyte differentiation, food intake, energy homeostasis, and cholesterol metabolism
– A response similar to that of humans to diet modifications (i.e., modulation of leptin and adiponectin, alteration of triglyceride levels, and hepatic steatosis)
– Accumulation of lipids in visceral, intramuscular, and subcutaneous adipocyte tissue
– Similar conservation, distribution, and formation of fat in adipose tissue to mammals
– Optical transparency
– Visualization of adipocyte formation and fatty acid uptake in vivo (via the use of colorants or fluorescent probes)
– External development
– Easy embryonic and genetic manipulation
– High identity in the genome for PPARs with mice and humans
– Easy modulation of microbiota
– Low maintenance cost of breeding
– Short life cycle and rapid reproduction rate
– Screening of compounds on a large scale
– Good intermediate between in vitro and murine models
**DISADVANTAGES**
– Lack of thermoregulation and brown adipose tissue
– The low similarity of leptin with humans (19%)
– Leptin receptor and UCPs proteins are not localized in adipose tissue
– Lack of protocols standardizing feeding and housing

**Table 2:** Anti-obesity effects of flavonoids in zebrafish.

Flavonoid	Bioactivity	Zebrafish Model	Reference
Rutin and quercetin	Reduction of fat reserve (40%); change in the expression of *mtp*, *fasn*, and *ctp1b*; remodeling of the fatty acid content and reduction in the *trans*-fatty acid content;	Zebrafish embryos	[71]
Glucoside derivatives of quercetin, kaempferol and isorhamnetin; rutin, procyanidin B1-B3; catechin and epicatechin; taxifolin; dihydrokaempferol; ploridizin	Lowering effect of BMI value; reduction of the area of adipose tissue, as well as both number and size of adipocytes; restoration of the levels of *ghrl* and *lep*	Overfed zebrafish	[74]
Silibinin	Downregulation of adipogenic factors *pparg, cebpa* and *fabp11a*	Zebrafish larvae fed with a high-fat diet	[78]
Baicalein	Reduction of lipid accumulation (30%); downregulation of adipogenic genes *pparg, cebpa, fabp2a/b* and *srbp1/2*	Zebrafish embryos fed with a high-fat diet	[79]
Kaempferol	Reduction of lipid droplets; suppression of expression of *pparg, cebpa, fabp2a/b* and *srbp1/2*	Zebrafish larvae fed with a high-fat diet	[81]
Quercetin	Reduction of triglyceride accumulation and NO generation	Zebrafish embryos fed with a high-fat diet	[83]
Lucenin-2; vicenin-2; lucenin-2-4′-methyl ester; eriocitrin; narirutin; hesperidin; sinensetin; nobiletin	Reduction of body weight, BMI value, and both the number and size of adipocytes; regulation of obesity-related genes *lep, ghrl, hcrt, pomc* and *npy*	Overfed zebrafish	[89]

**Table 3:** Health-promoting effects of flavonoids against obesity-related metabolic diseases in zebrafish.

Flavonoid	Bioactivity	Obesity-Related Metabolic Disease	Zebrafish Model	Reference
Eriocitrin	Improvement of dyslipidemia; reduction in lipid droplets in the liver; activation of mitochondrial biogenesis	NAFLD	Zebrafish with diet-induced obesity	[96]
Isobavachalcone	Reduction of intrahepatic fat deposits; improvement of liver steatosis; downregulation of *cepba* and *pparg*	Steatosis related to obesity	Zebrafish fed with a high-fat cholesterol diet	[97]
Nobiletin	Reduction of ANGPTL3 protein expression; lowering of plasma levels of triglycerides and cholesterol	Dyslipidemia and atherosclerotic cardiovascular diseases	High-fat diet-fed zebrafish	[102]
Lucenin-2; vicenin-2; lucenin-2-4′-methyl ester; eriocitrin; narirutin; hesperidin; sinensetin; nobiletin	Reduction of tissue inflammatory events; downregulation of proinflammatory cytokine genes *il1b*, *il6* and *tnfa*	Enteritis	Adult zebrafish subjected to *Vibrio anguillarum*-induced enteritis	[103]
Apigenin and quercetin	Increase of superoxide dismutase and catalase enzymes; downregulation of *ptgs2a*, *il1b* and *tnfa*; upregulation of *il10*; activation of MAPKs (ERK_1/2_ and p38); suppression of NF-κB pathway	Intestinal inflammation	Zebrafish model exposed to k-carrageenan	[104]
Linarin, diosmetin-7-glucoside and tilianin	Inhibition of expression of *il1b*, *il8* and *mmp9* and stimulation of the superoxide dismutase activity	IBD	Zebrafish larvae exposed to DSS	[106]
Leptosidin, leptosin, isoquercetin, and astragalin	Protective effect on the pancreatic islets damaged by alloxan	Insulin resistance and type 2 diabetes	Zebrafish subjected to alloxan	[107]

## Data Availability

Not applicable.
